# Supplementary material for: Erythrocyte Membrane Cloaked Curcumin-Loaded Nanoparticles for Enhanced Chemotherapy
Source: Pharmaceutics. 2019 Aug 23;11(9):429. doi: 10.3390/pharmaceutics11090429 (PMC6781301; doi:10.3390/pharmaceutics11090429)
Supplement: Supplementary file 1 [file pharmaceutics-11-00429-s001.pdf]

pharmaceutics

Supplementary Materials: Erythrocyte Membrane Cloaked Curcumin-Loaded Nanoparticles for Enhanced Chemotherapy

Xiaotian Xie, Haijun Wang, Gareth R. Williams, Yanbo Yang, Yongli Zheng, Junzi Wu and Li-Min Zhu

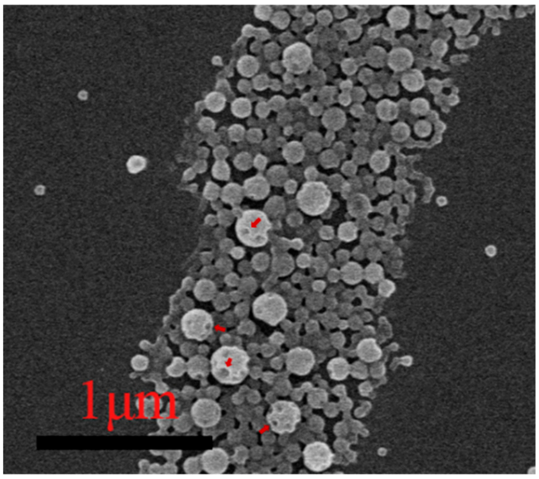

Figure S1. A FESEM images of porous PLGA NPs prepared at 300 rpm. Pores are marked with red arrows.

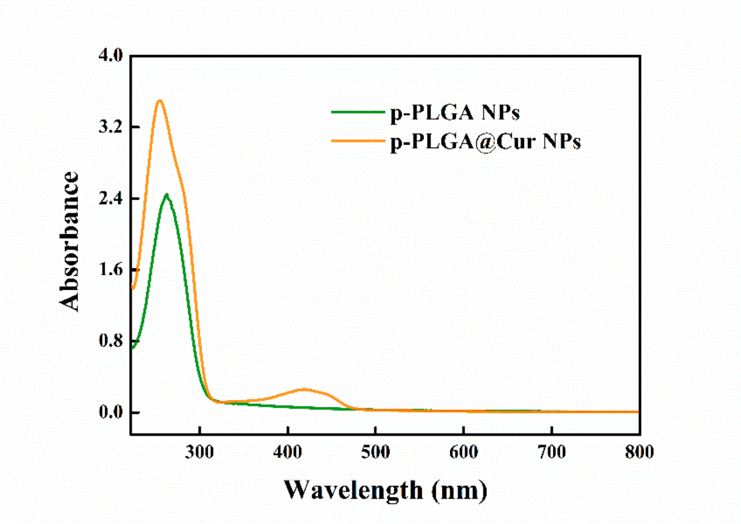

Figure S2. UV-vis spectra of p-PLGA and p-PLGA@Cur NPs.

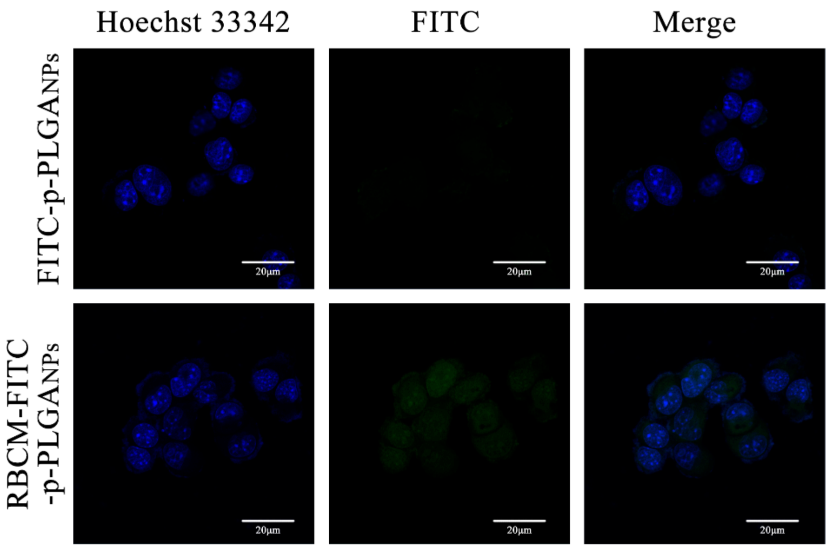

Figure S3. CLSM images of 4T1 cells exposed to FITC-p-PLGA and RBCM-FITC-p-PLGA NPs. Representative images from three independent experiments are shown.

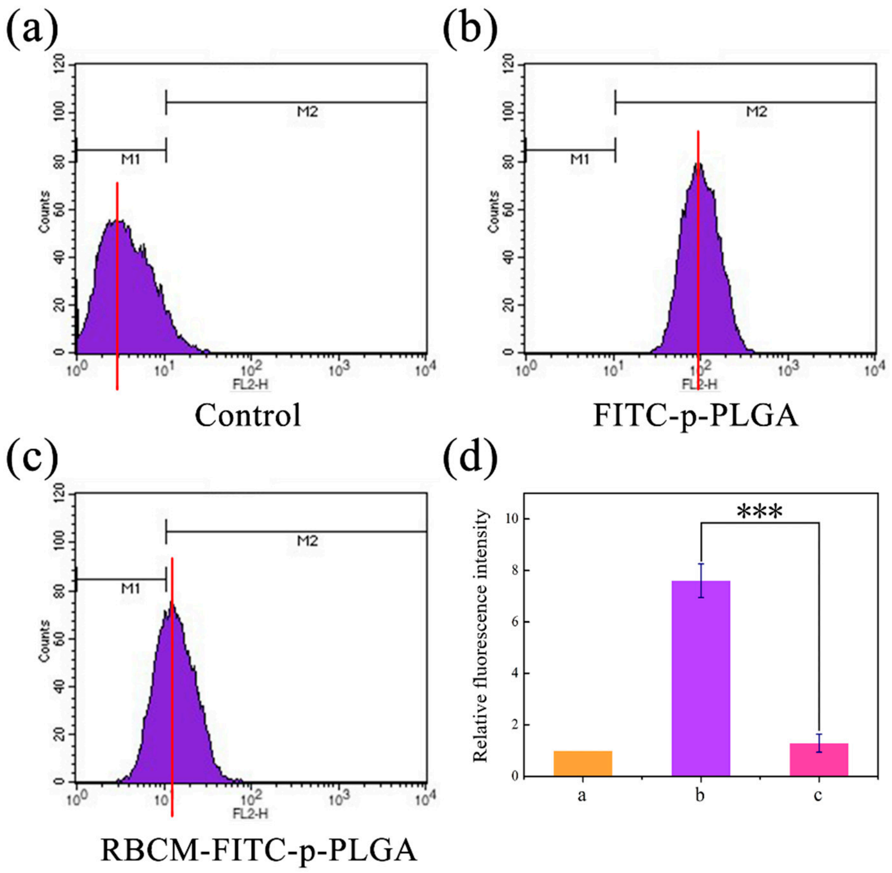

Figure S4. Flow cytometry data for: (**a**) untreated RAW 264.7 cells and RAW 264.7 cells incubated for 4 h with FITC labeled (**b**) p-PLGA NPs or (**c**) RBCM-p-PLGA NPs. (**d**) The relative fluorescence intensity values calculated from panels (**a**)–(**c**). The data are represented as mean ± S.D. (n = 3). *** p < 0.001.

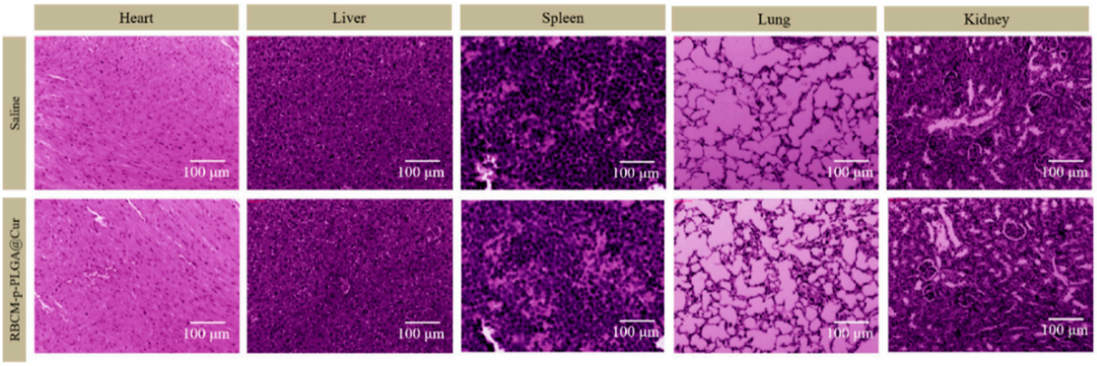

Figure S5. H&E staining images (100×) of the major organs from H22 tumor-bearing mice after treatment with saline or RBCM-p-PLGA@Cur NPs.

Table S1. The results of Peppas analysis of the drug release data.

Sample	T = 37 °C
PLGA@Cur	$Q = 6.848t^{0.389}$ ($R^2 = 0.9920$)
p-PLGA@Cur	$Q = 11.898t^{0.606}$ ($R^2 = 0.8643$)
RBCM-p-PLGA@Cur	$Q = 8.141t^{0.595}$ ($R^2 = 0.9783$)
